# Does the Cortical-Depth Dependence of the Hemodynamic Response Function Differ Between Age Groups?

**DOI:** 10.1007/s10548-025-01107-0

**Published:** 2025-02-28

**Authors:** Luisa Raimondo, Jurjen Heij, Tomas Knapen, Jeroen C. W. Siero, Wietske van der Zwaag, Serge O. Dumoulin

**Affiliations:** 1 Spinoza Centre for Neuroimaging, Meibergdreef 75, 1105 BK Amsterdam, The Netherlands; 2 Computational Cognitive Neuroscience and Neuroimaging, Netherlands Institute for Neuroscience, Amsterdam, The Netherlands; 3 Experimental and Applied Psychology, VU University, Amsterdam, The Netherlands; 4 Experimental Psychology, Utrecht University, Utrecht, The Netherlands; 5 Radiology, University Medical Centre Utrecht, Utrecht, The Netherlands

**Keywords:** Line-scanning, BOLD fMRI, 7 T, HRF, Aging, Deconvolution

## Abstract

Functional magnetic resonance imaging (fMRI) is a widely used tool to investigate the functional brain responses in living humans. Valid comparisons of fMRI results depend on consistency of the blood-oxygen-level-dependent (BOLD) hemodynamic response function (HRF). Although common statistical approaches assume a single HRF across the entire brain, the HRF differs across individuals, regions of the brain, and cortical depth. Here, we measure HRF properties in primary visual cortex (V1) using 7 T fMRI with ultra-high spatiotemporal resolution line-scanning (250 μm in laminar direction, sampled every 105 ms). Line-scanning allowed us to investigate age-related HRF changes as a function of cortical depth. Eleven young and eleven middle-aged healthy participants participated in the experiments. We estimated the HRFs using a smooth basis function deconvolution approach. We also compared the results with conventional resolutions. From these HRFs, we extracted properties related to response magnitude and temporal dynamics. The cortical depth dependent HRFs were similar to the HRFs extracted using conventional resolutions validating the cortical depth dependent approach. We found that the properties of the HRF in the two age groups are similar across cortical depth. In other words, the variance between participants is larger than the variance between age groups. This suggests that middle-aged individuals can participate in cortical depth dependent studies free of bias in HRF properties.

**Supplementary Information:**

The online version contains supplementary material available at 10.1007/s10548-025-01107-0.

## Introduction

Aging causes a broad set of anatomical changes in the brain, such as thinning and atrophy of the cortex (Salat et al. 2004). Aging also changes function, such as a reduction in cerebral blood flow (CBF), alterations in cerebral metabolic rate of oxygen (CMRO_2_) and blood supply (Chen et al. 2011; Lu et al. 2011). Magnetic resonance imaging (MRI) is a powerful tool, since it allows for measurements of structure and function in the living human brain. Particularly, functional MRI (fMRI) is a non-invasive method that is sensitive to changes in the blood oxygenation level-dependent (BOLD) signal as a consequence of tasks (Ogawa et al. 1990). With respect to aging, fMRI can detect changes in the hemodynamic response function (HRF), which may reflect changes in the vasculature, particularly when sufficient high spatiotemporal resolution is employed.

Several studies investigated the age-dependence of fMRI responses, but there is no clear consensus on whether, and if so how, the HRF changes in healthy aging. On the one hand, some studies reported a decrease in the amplitude of the BOLD response following a visual stimulus in older individuals compared to a younger group (66–89 and 18–24 years old and 57–84 and 20–36 years old) (Buckner et al. 2000; Ross et al. 1997). On the other hand, Huettel et al. (Huettel et al. 2001) found similar BOLD response amplitudes. West et al. (West et al. 2019) suggested the small sample sizes, analysis techniques, and physiological mechanisms could cause between-study discrepancies. Using large sample sizes and minimal analysis assumptions, they aimed to disentangle age-related changes in HRF parameters from other sources of variability and link them solely to alterations in one or more components of the neural-vascular coupling system. This approach yielded a delayed response with decreased amplitude, reduced undershoot and longer return to baseline in elderly (54–74 years old) compared to younger (18–30 years old) participants in the occipital cortex in response to a visual-motor task. Moreover, the older group exhibited higher variability in response shape compared to the younger group. However, in an earlier study, a lower amplitude and SNR were not linked to shape and or variability differences between age groups (D’Esposito et al. 1999).

Despite the discrepancies reported above, some common points of those studies can be highlighted: relatively low magnetic fields (1.5 and 3 T), low spatial and temporal resolutions (> 3 mm isotropic) and long repetition times (TR) of 1–2 s were used, which were standard for fMRI at the time (Logothetis 2008; Turner 2016). These acquisitions are thus likely biased to sample BOLD signals from larger, draining veins (Turner 2002). In contrast, contemporary hardware (e.g. UHF-MRI) and methodological (modeling approaches) advances are likely to bring relevant insights in HRF characterization (Chen et al. 2021), which can be relevant for the aging brain. Particularly, increased field strengths can be employed to reach higher spatial and temporal resolution. This, in turn, would allow a more spatially localized detection of changes in the HRF through reduction of partial volume effects (Dumoulin et al. 2018; Raimondo et al. 2021b; Siero et al. 2011; van der Zwaag et al. 2016). Using a higher sampling rate allows more frequent sampling along the HRF, which aids in estimation of shape and timing parameters.

This study aims measure age-related changes in HRFs across cortical depth using line-scanning fMRI. In contrast to the conventional whole-brain acquisition, line-scanning is based on the acquisition of one dimensional data, to reach extremely high spatial (250 μm) and temporal (~ 100 ms) resolution along the line direction (Choi et al. 2021; Raimondo et al. 2021a; Yu et al. 2014). This is done by exciting a slice and suppressing the signal outside the line of interest through outer volume suppression (OVS). The phase-encoding gradient in the direction perpendicular to the line is omitted, and the line signal is then acquired after every excitation pulse. This method is suitable to yield highly detailed HRFs in humans across cortical depth by positioning the line perpendicular to the cortex, thereby reducing the mixing of signals from different cortical depths. We also included a relatively high-resolution whole-brain fMRI acquisition (1.8 mm isotropic spatial resolution and repetition time of 1.32 s). The same visual task was performed in both acquisition schemes. We selected two age groups (19–25 years old, denoted ‘young’ and 57–69 years old, denoted ‘middle aged’) and we extracted the HRF for a large ROI in the primary visual cortex (V1) from the whole brain data acquisition and HRFs obtained at different depths from line-scanning data.

## Materials and Methods

### Participants

11 young healthy participants (23 ± 2 years old, age range 19–25, 6 males, 5 females) and 11 middle aged healthy participants (63 ± 4 years old, age range 57–69, 9 males, 2 females) were scanned with a 7 T MRI system (Philips, Netherlands) equipped with a 2-channel transmit and 32 channel receive head coil (Nova Medical, USA). Following the Helsinki Declaration, all participants provided written informed consent before participating. This study was approved by the local ethical committee of the Vrije Universiteit Amsterdam. Participants were screened prior to the experiments to ensure MR compatibility. We carefully instructed participants to lay still in the scanner and motion was limited by fixing the head using foam pads. We excluded data from 2 participants of the “middle aged” group from the line-scanning dataset. One due to misplacement of the line (i.e. the line was positioned far from the probabilistic V1) and another due to excessive motion.

### Stimulus and Task

**Fig. 1:**
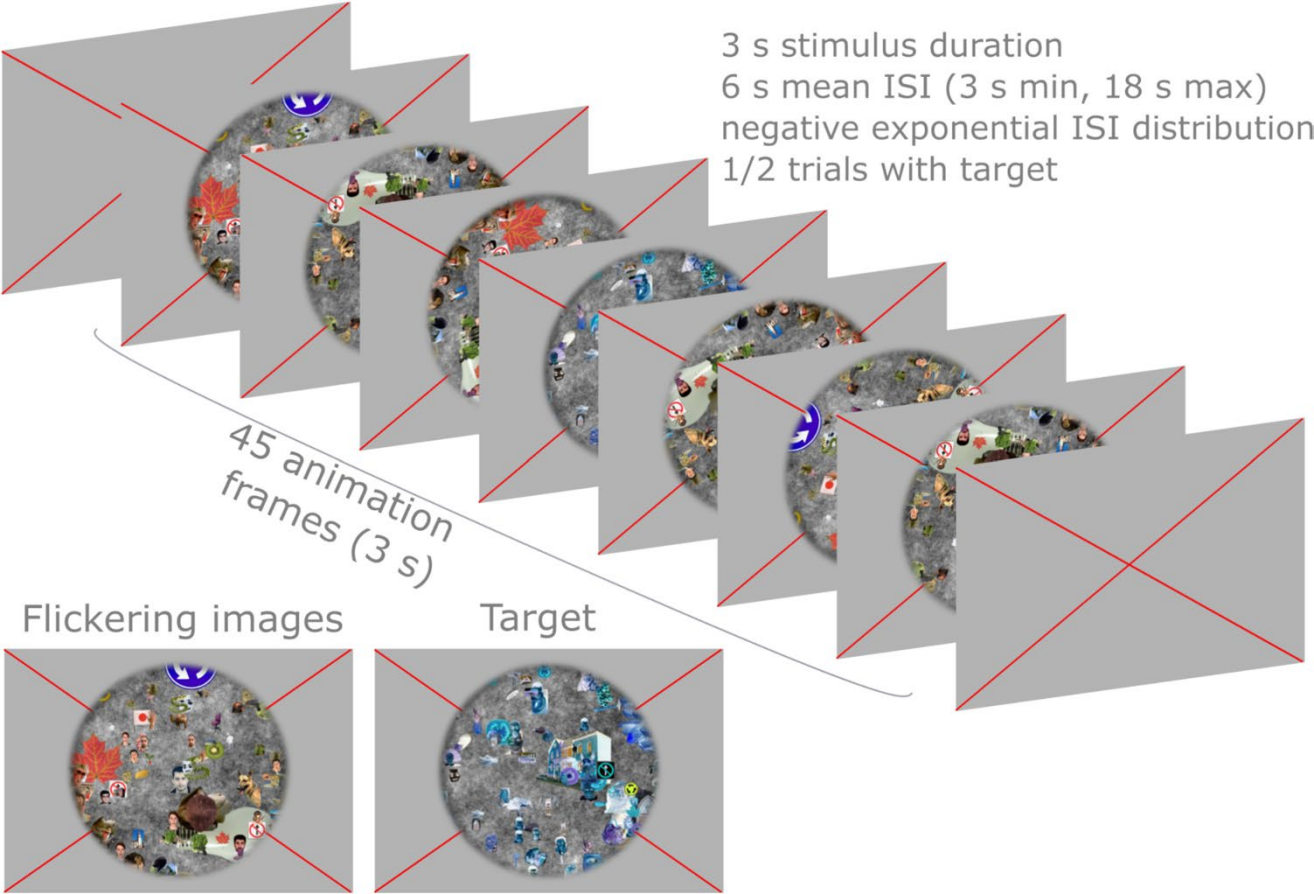
Schematic representation of the event-related visual stimulus. Images selected from the HCP retinotopy dataset (Benson et al. 2018) were flickering at 15 Hz on a scrambled gray background, in an event-related manner: 3 s stimulus duration (i.e. 45 animation frames), 6 s mean inter-stimulus interval (ISI), 3 s minimum ISI, 18 s maximum ISI, following a negative exponential ISI distribution. Images were vignetted with a circular aperture subtending 10° of visual angle. In half of the trials negative images (target) were introduced to keep the participants focused. The participants were instructed to press a button whenever a target was displayed

The visual stimulus was presented on a screen placed at the end of the scanner bore, visible through a mirror positioned on the top of the coil. To independently localize responses in the visual cortex that correspond to our stimulus extent (Maus et al. 2010), we presented a 12 s ON/OFF “localizer” task of 2.5 min. During the ON-period, objects flickering at 8 Hz were presented on a scrambled gray background, while during the OFF-period a gray screen was displayed. For estimation of HRF shapes (Birn et al. 2002; Friston et al. 1999; Liu et al. 2001), we employed an event-related visual task (Fig. 1), consisting of flickering images at 15 Hz on a scrambled gray background, in an event-related manner [for stimulus details, see Benson et al. (2018)]. Whereas Benson, et al. used apertures to elicit retinotopic responses, we vignetted the images only with a circular aperture subtending 10 degrees of visual angle. Based on the frequency (15 Hz) and duration (3 s), 45 images per trial were randomly selected. This dense number of unique stimuli ensures an even activation across large portions of visual cortex. The inter-stimulus intervals (ISIs) were jittered following a negative exponential decay to reduce collinearity between subsequent events (Chen et al. 2023; Friston et al. 1999; Mumford et al. 2015). We optimized the ISI distribution in two stages. First, we generated 1000 ISI distributions (ISI_min_/ISI_max_/ISI_mean_ = 3 s/18 s/6 s) and obtained the predicted time course using a canonical HRF and a stimulus duration of 3 s. The ISI distribution that resulted in the prediction with the highest variance (ISI_optimized_) was entered in stage 2. Here, we optimized the order of ISIs by selecting the predicted time course with the highest variance generated from all possible ISI orders in ISI_optimized_. To ensure participants’ engagement, we introduced contrast-inverted presentations of flickering images (*target*) lasting ~ 0.3 s in half of the trials and instructed participants to press a button whenever they detected a target. The target could be anywhere in the stream of images by randomly selecting an index between 0 and 45; the contrast of subsequent images lasting the duration was inverted. Depending on the performance outside the scanner, we adjusted the duration of the target. A baseline consisting of 20 s of gray screen was added in the beginning of the task.

The full experiment code can be found at the following link: https://github.com/gjheij/LineExps/tree/main/scenes*.*

### Data Acquisition

We acquired anatomical scans with an MP2RAGE sequence (magnetization-prepared 2 rapid acquisition gradient echo) (Marques et al. 2010; Oliveira et al. 2021): matrix size = 344 × 344, spatial resolution = 0.64 mm isotropic, TR = 6.2 ms, TE = 2.3 ms, TI_1_/TI_2_ = 0.8/2.7 s, flip angle = 8°/5°. A fluid-attenuated inversion recovery (FLAIR) sequence with the following parameters was acquired to aid in pial surface segmentation: matrix size = 220 × 220 spatial resolution = 1 mm isotropic, TR/TI = 8/2.2 s, TE = 234 ms, flip angle = 90°.

**Fig. 2:**
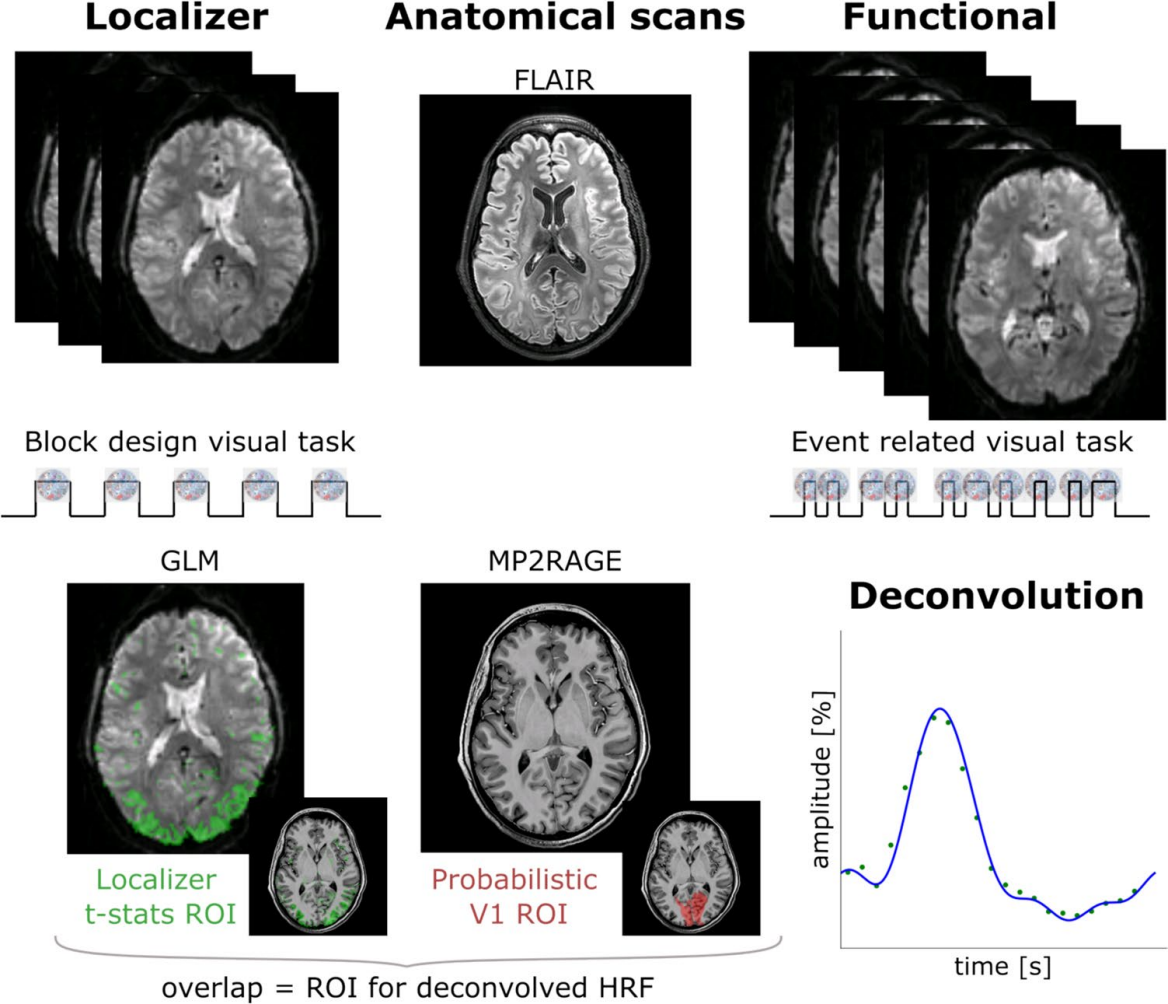
Schematic representation of the whole brain data acquisition and analysis. From the localizer with the block design task, a localizer ROI was created with voxels with t-stats > 2.3 following the GLM analysis. A binary V1 ROI (pV1 ROI) was derived from the Juelich Histological Atlas (Amunts et al. 2000) (threshold = 39%) as implemented in FSL (Smith 2004) and transformed to the functional space. The overlap of localizer ROI and pV1 ROI resulted in voxels eligible for the deconvolution approach from the functional runs with event-related task

Whole brain functional data were acquired using a 3D GE-EPI sequence with the following parameters: matrix size = 112 × 112, spatial resolution = 1.8 mm isotropic, TR = 1.32 s, TE = 17 ms, flip angle = 13°, SENSE_PE_ = 2.61, SENSE_slice_ = 3.27. Each run was followed by 4 volumes acquired in the opposite phase-encoding direction to correct for susceptibility distortions (Andersson et al. 2003). The localizer run consisted of 124 volumes (2.5 min), while 230 volumes per event-related run were acquired (5 min 11 s). Note that for the whole-brain acquisitions, the ISI distribution was different for every run. Right before the line-scanning acquisitions, we acquired a fast 3D FFE T1-weighted anatomical scan (matrix size = 124 × 124 with 87 slices, spatial resolution = 2 mm isotropic, TR/TE = 3.7/1.7 ms, TFE factor = 352, flip angle = 7°, acquisition time = 56 s) that was used as an intermediate image to register the 2D slice to the MP2RAGE. A schematic representation of the overall whole brain data acquisition can be seen in the top part of Fig. 2.

### Data Acquisition: Line-Scanning Data

**Fig. 3:**
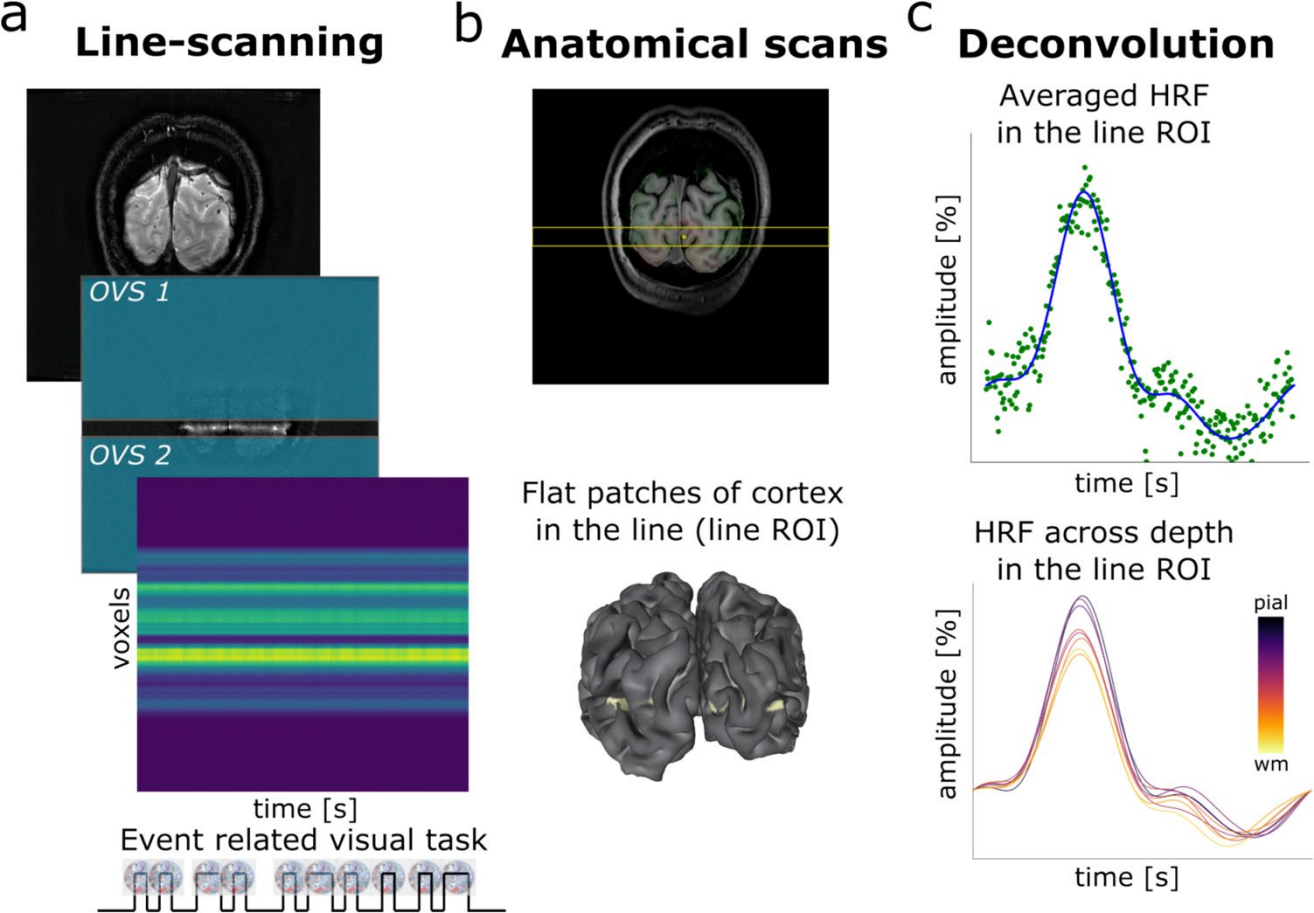
Schematic representation of line-scanning data acquisition and analysis. **a** A gradient-echo slice with and without OVS bands was acquired, followed by the line-scanning runs with the event-related visual task. **b** The slice was coregistered to the whole brain anatomical scan (MP2RAGE) and a patch of cortex relatively perpendicular to the line was selected (line ROI). **c** The deconvolved HRF was extracted across cortical depth along the line ROI, as well as averaged across the line ROI

The line-scanning acquisition was based on (Raimondo et al. 2023, 2021a). First, a single slice gradient-echo acquisition (matrix size = 720 × 180, in-plane spatial resolution = 0.25 × 1 mm^2^, slice thickness = 2.5 mm, TR = 106 ms, TE = 12 ms, flip angle = 22°) was done to identify the target area. Second, a line-signal distribution (LSD) image was acquired which was identical to the slice acquisition, with the addition of 2 saturation pulses (OVS bands, 7.76 ms pulse duration) to suppress the signal outside the line of interest. Finally, 3 runs of functional line-scanning data were acquired using a modified 2D multi-echo gradient-echo sequence (5 echoes), where the phase-encoding in the direction perpendicular to the line, needed for conventional 2D imaging, was omitted. Specific parameters were as follows: line resolution = 250 μm, TR = 105 ms, TE_1_ = 6 ms, ΔTE = 8 ms, flip angle = 16°, array size = 720, line thickness = 2.5 mm, in-plane line width = 4 mm, fat suppression using SPIR. The 2 saturation pulses were identical to those used in the LSD image. The left part of Fig. 3 shows a schematic representation of the line-scanning data acquisition.

Regarding the planning procedure, a single slice was positioned coronally, crossing the visual cortex (Fig. 3). By design, the line is located in the middle of the slice. Here, the slice was placed so that the line crossed the occipital lobe in the V1 area, approximately in a straight patch of cortex, at a location corresponding to a pRF peripheral (~ 1 dva) in the visual field. The line was always positioned in the left–right direction. This places the line approximately perpendicular to the medial gray matter sheet of the occipital lobe, which helps to avoid partial volume effects. We tried to avoid voxels which clearly included large veins, which are visible as black dots on the single slice gradient-echo acquisition.

### Estimation of the HRF Shape Using Deconvolution

Deconvolution was performed using Nideconv (https://github.com/VU-Cog-Sci/nideconv). A finite impulse response model with a regressor per TR (time period/TR) was applied to capture shapes without any assumptions regarding HRF shape. The time period included 3 s before stimulus onset until 27 s after stimulus onset. The large number of regressors used with FIR inflates variability, as fewer samples contribute to each parameter estimate (Poldrack et al. 2011). To deal with this, we complemented the analysis with a deconvolution using a Fourier basis set. Such an approach imposes some constraints on HRF shape (such as its intrinsic low-pass filtering of the resulting shape by excluding high frequencies from the response shape basis set), but is still flexible because different linear combinations allow for larger variety of HRF shapes than a single canonical basis function (Poldrack et al. 2011). We used a cross-validation framework to derive the number of regressors that maximized out-of-set variance explained, resulting in 10 regressors (Fig. S1). The following parameters were derived from the response estimations: response magnitude (amplitude), time-to-peak, full-width-at-half-maximum (FWHM), rising slope (i.e. the angulation of the rising part of the HRF; evaluated from the derivative), positive area under the curve, and post-stimulus undershoot area.

### Data Analysis: Whole Brain Data

The T_1_-weighted (T1w) anatomical image from the MP2RAGE sequence was processed as follows: first, the image was denoised using a spatial-adaptive Non-Local Means (SANLM-) (Manjón et al. 2010) and segmented into cerebrospinal fluid (CSF), white-matter (WM) and gray-matter (GM) using CAT12 (https://neuro-jena.github.io/cat/). The denoised image was corrected for intensity non-uniformity with N4BiasFieldCorrection (Tustison et al. 2010), distributed with ANTs 2.3.3 (https://github.com/ANTsX/ANTs). A mask representing the sagittal sinus was created by hand using ITK-Snap (Yushkevich et al. 2016, 2006).The voxels in the mask were set to zero in the denoised T1w image to limit the necessity for manual intervention after surface reconstruction. The final masked image was then skull-stripped with a Nipype implementation of the antsBrainExtraction.sh workflow (from ANTs), using OASIS30ANTs as target template. Brain tissue segmentation of CSF, WM and GM was performed on the brain-extracted T1w image using FSL’s *FAST* (Zhang et al. 2001). FreeSurfer 7.2 recon-all (Dale et al. 1999) was used to obtain native cortical surface reconstructions. The software makes use of the FLAIR image to refine the segmentation obtained by T1w image alone, particularly in the exclusion of sinus and at the pial surface border. We defined a V1 region of interest (pV1 ROI) on the anatomical data, using FSL, by binarizing the probabilistic V1 ROI from the Juelich histological atlas (Amunts et al. 2000), thresholded by 39%. The data from the block design localizer were analyzed with a general linear model (GLM) analysis, assuming a Gaussian HRF shape and t statistical values (t-stats) were estimated. The voxels with t-stats ≥ 2.3 (p-value 0.001) were used to make a localizer ROI. The time courses from overlapping voxels of the pV1 ROI and localizer ROI were converted in percentage signal change and averaged, from the baseline at the beginning of the visual task.

### Data Analysis: Line-Scanning Data

The reconstruction of line-scanning data was performed offline using MatLab, Gyrotools. We combined the multi-channel coil data with a temporal signal-to-noise ratio (tSNR) and coil sensitivity-weighted sum of squares (SoS) weighted scheme per echo as in (Raimondo et al. 2023). Prior to channel combination, we applied a NOise reduction with DIstribution corrected PCA (NORDIC) denoising step, while multi-echo data were combined with a sum of squares (Raimondo et al. 2023; Vizioli et al. 2021). We averaged the 3 runs of line-scanning data to increase the signal-to-noise ratio (SNR), akin to (Cai et al. 2021).

Single slice images were registered to the MP2RAGE scan via the fast anatomical scan. To select the area of the line most perpendicular to the cortex, we projected the line image to the surface and identified the point with the smallest angle between the coronal vector (the line direction) and the normal vector to the cortex. The voxels surrounding the most perpendicular vertex were selected to cover the pial surface to the white matter boundary to form the line ROI. The Supplementary material (Fig. S2) shows each participant's line ROI after coregistration to the anatomical MP2RAGE. We also highlighted on the same slices the pV1 ROI and the localizer ROI to confirm that the line ROI ended up in an active region of V1 for each participant. Since the number of voxels that we selected for the line ROI differed across participants, we converted the number of voxels to percentage of distance from the cortex in order to pool the values of the HRF parameters across participants. We also averaged all the HRFs across cortical depth for a closer comparison with whole brain data in visual cortex. Note that the post stimulus undershoot was computed only for the averaged data across cortical depth, since the single HRF for each cortical depth was too noisy to perform this estimation.

## Results

### Participants in Both Age Categories were Able to Perform the Task

**Fig. 4:**
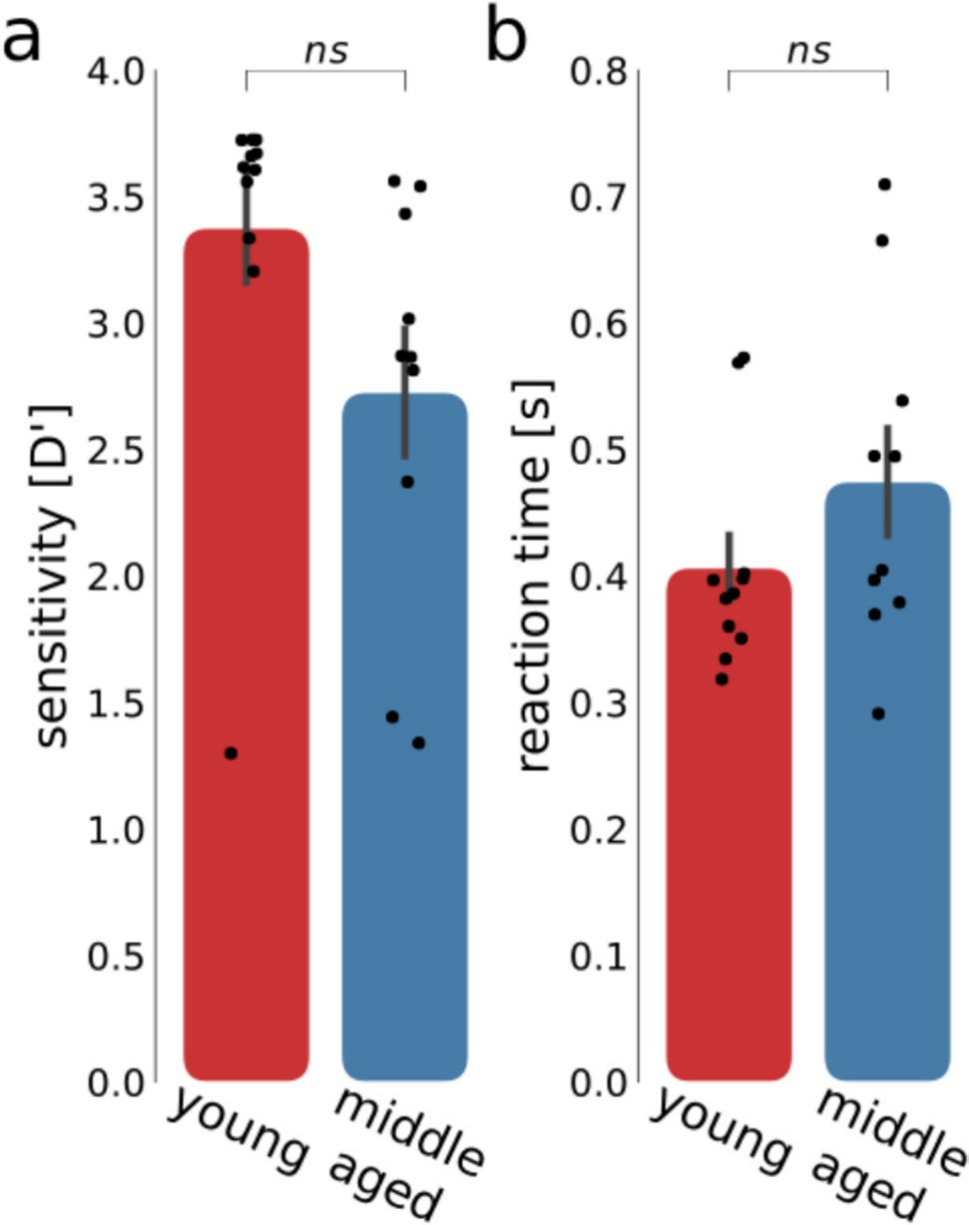
**a** d-prime values for all participants in the two age groups (red for young and blue for middle-aged). **b** reaction times for all participants in the 2 age groups (red for young and blue for middle-aged). All d’ values were larger than 1 indicating that the participants were able to do the task. Moreover, no significant differences were observed between age-groups

We evaluated the ability of the participants to perform the task, i.e. press the button when the contrast-inverted images (*target*) were presented, by calculating the average discrimination index (d’) and their reaction time across runs (See Fig. 4 for individual participant performance, apart from one participant of the middle-aged group, for which no response was recorded due to technical problems). First, all participants had d’ values above 1 indicating that all participants were able to discriminate the target image and performed the task according to the instructions. Second, we compared performance between age-groups. The participants performed with d’ values of 3.4 ± 0.7 (mean ± standard deviation) for the young group and 2.7 ± 0.8 (mean ± standard deviation) for the middle-aged group, while the averaged reaction times were 0.41 ± 0.09 s (mean ± standard deviation) for the young group and 0.47 ± 0.13 s (mean ± standard deviation) for the middle-aged participants. Neither d-prime values, nor reaction time were significantly different in the 2 age groups when performing a Student’s *t*-test. Therefore, both age-groups paid attention to the stimulus and were able to perform the task. Differences in task performance are not likely to underlie potential differences in the HRFs.

### Similar HRF Shapes Between Age-Groups Extracted from pV1

**Fig. 5:**
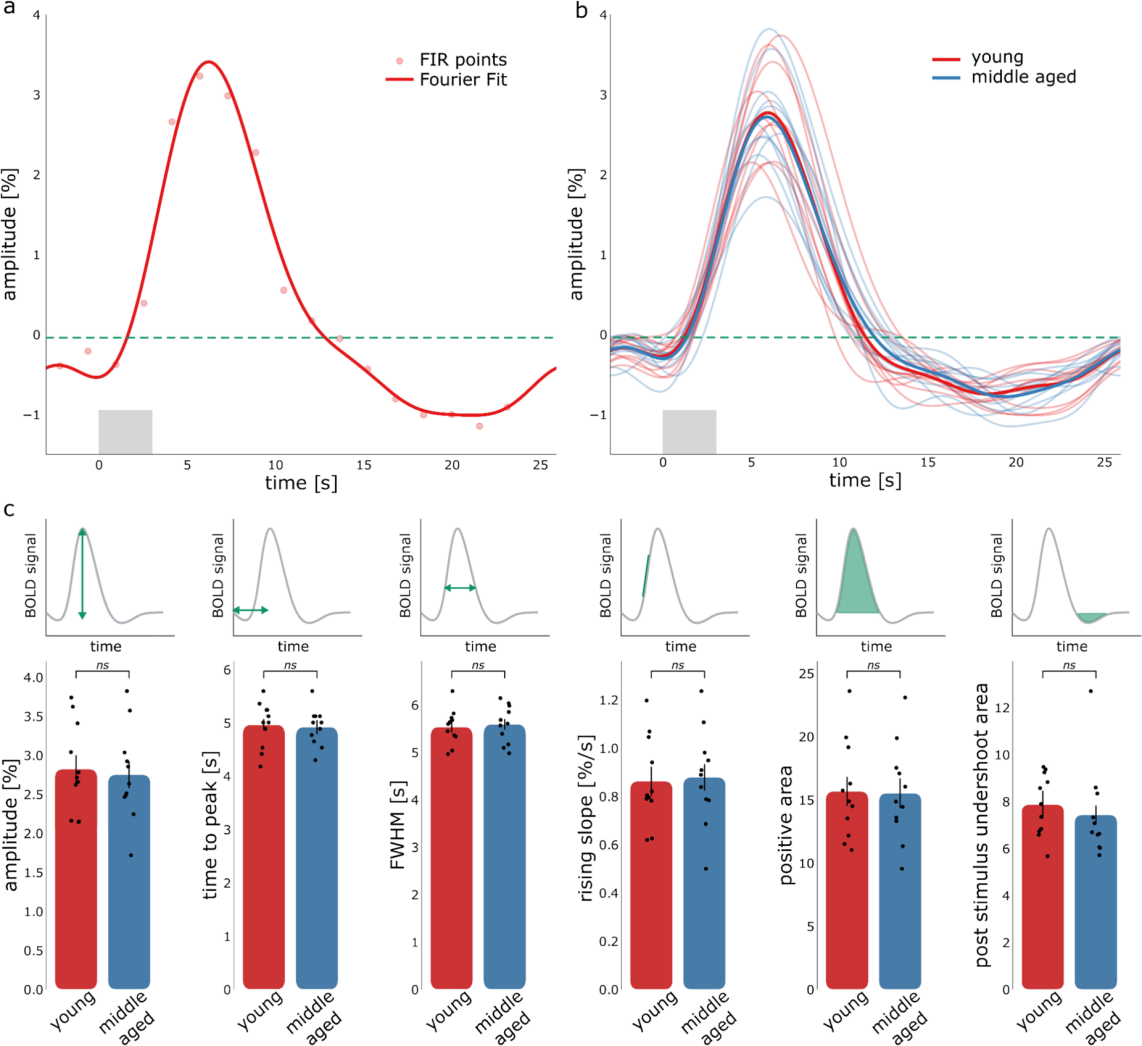
**a** Extracted HRF from pV1 (with FIR points and Fourier basis set), for a representative participant. **b** HRFs for all participants in the young (red) and middle-aged (blue) group (thin lines) and averaged HRF for both groups (thick lines). The light gray box indicates the period when the task was shown. **c** HRF parameters from whole brain data for the 2 age groups (red young and blue middle aged). The error bar indicates the standard error of the mean. We found no significant differences for the HRF parameters between age groups

Figure 5 shows the deconvolved HRFs extracted from the pV1 ROI. The deconvolved HRF using Fourier basis sets is superimposed on the points from the FIR model for a representative participant (for all individual participants please see supplementary material Fig. S3), showing good agreement between the Fourier basis sets and FIR model. The deconvolved HRFs are shown together for all participants in Fig. 5b (light lines), along with the averages (darker lines) for the young (red) and middle-aged group (blue).

Group-average HRF shapes between age-groups were remarkably similar, as confirmed by the analysis of the main HRFs parameters (Fig. 5c). No significant differences (Student’s *t*-test) were found for amplitude (t_20_ = − 0.287, *p* = 0.777), time to peak (t_20_ = − 0.257, *p* = 0.800), FWHM (t_20_ = 0.339, *p* = 0.738), rising slope (t_20_ = 0.199, *p* = 0.845), positive area under the curve (t_20_ = − 0.088, *p* = 0.931), and post stimulus undershoot area (t_20_ = 0.611, *p* = 0.538). Furthermore, we compared the HRF parameters among the 2 age groups using a Bayesian independent samples t-test to estimate evidence in favor of the alternative hypothesis (i.e. parameters being different). We found moderate-weak evidence that all the parameters are identical across groups (BF_10,amplitude_ = 0.396, BF_10,time-to-peak_ = 0.394, _BF10, FWHM_ = 0.401, BF_10, rising slope_ = 0.39, BF_10, positive area_ = 0.386, BF_10, post stimulus undershoot area_ = 0.44).

### Average HRF Parameters Across Cortical Depth from Line-Scanning Data

Before analyzing line-scanning data we checked the results of the line planning procedure by plotting the pV1 mask on the slice on which the line was acquired, together with the localizer ROI from the whole brain functional localizer (Fig. S2 of the Supplementary material). We selected the voxels along the line crossing the cortex from pial surface to WM boundary, as perpendicular as possible (line ROI). The angle between line and surface was calculated a posteriori, and was on average (18 ± 12)° for the young group and (22 ± 6)° for the middle aged group).

**Fig. 6:**
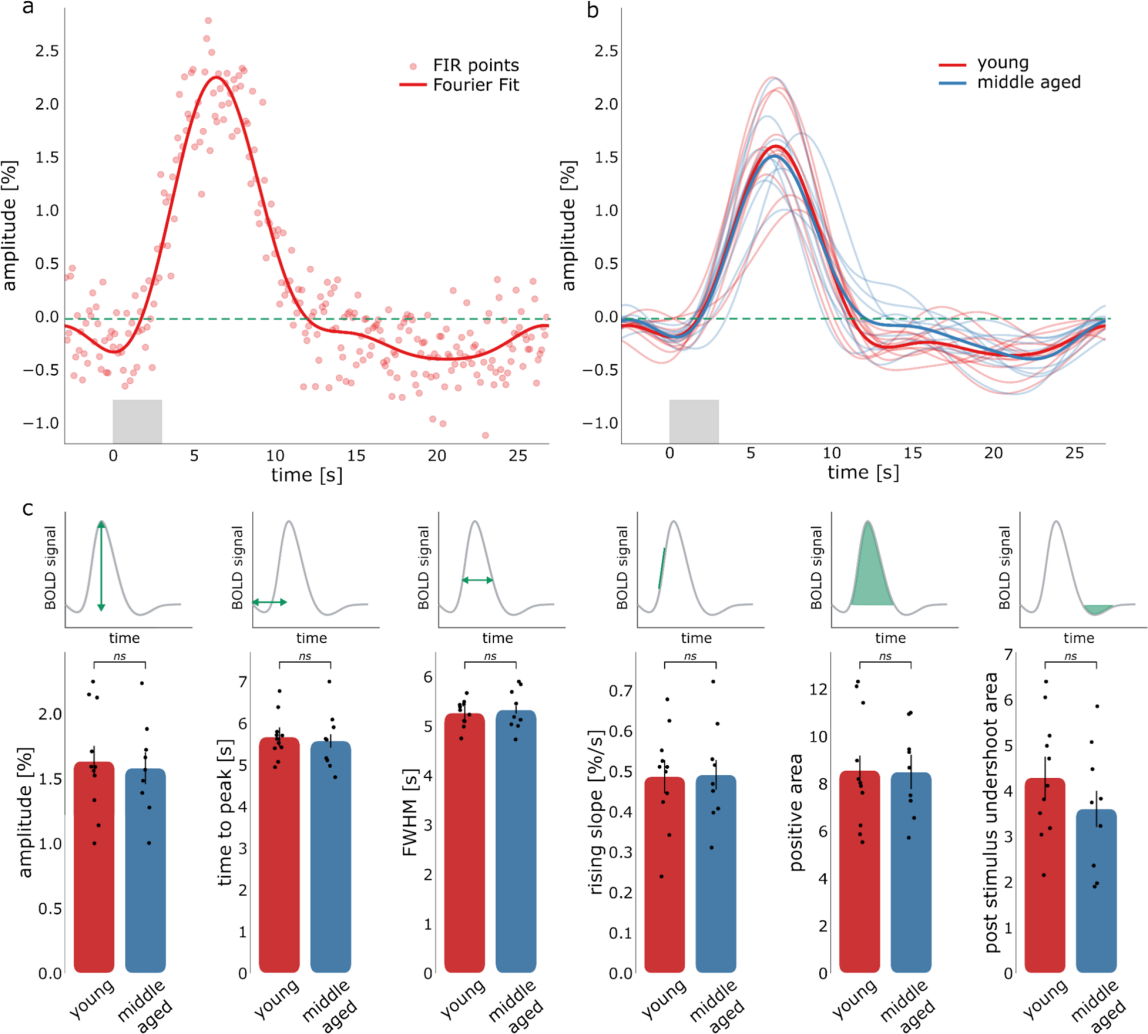
**a** Extracted HRF from line-scanning data averaged across cortical depths (with FIR points and Fourier basis set), for a representative participant. **b** HRFs averaged across cortical depth for all participants (red young group and blue middle aged group) and respective averaged HRF across the 2 age groups (ticker red and blue lines). The light gray box indicates the period when the task was shown. **c** HRF parameters averaged across cortical depth line-scanning data for the 2 age groups (red young and blue middle aged). Note that the line-scanning data for 2 participants were excluded from plot (**b**) and (**c**) as well as from the analysis

Figure 6 follows a similar layout compared to Fig. 5, but results are derived from the line-scanning acquisition. Figure 6a shows the deconvolved HRF for the same representative participant as Fig. 5a, together with the FIR points. Note the drastic increase in samples compared to the whole brain data in Fig. 5. This is because the TR used for line-scanning is ~ 13 times shorter than the TR of whole brain data acquisition (0.105 s vs 1.32 s). In the Supplementary material (Fig. S4) the deconvolved HRF with FIR points is reported for individual participants. The deconvolved HRFs are also represented for all participants in Fig. 6b, together with the averages for the age groups (with the same color coding as in Fig. 5).

The deconvolved HRFs follow similar trends, but they present slightly more variability compared to whole brain data. Again, no differences between age groups were found for amplitude (t_18_ = − 0.308, *p* = 0.762), time-to-peak (t_18_ = − 0.345, *p* = 0.734), FWHM (t_18_ = 0.429, *p* = 0.673), rising slope (t_18_ = 0.085, *p* = 0.933), positive area (t_18_ = − 0.070, *p* = 0.945), and post stimulus undershoot area (t_18_ = − 1.131, *p* = 0.273). When comparing the spatiotemporal HRF parameters among age groups with a Bayesian independent samples t-test, we found weak-moderate evidence that the parameters are identical between groups (BF_10, amplitude_ = 0.412, BF_10, time-to-peak_ = 0.416, BF_10, FWHM_ = 0.426, BF_10, rising slope_ = 0.4, BF_10, positive area_ = 0.399, BF_10, post stimulus undershoot area_ = 0.621).

### Line-Scanning Versus 3D-EPI Acquisition

**Fig. 7:**
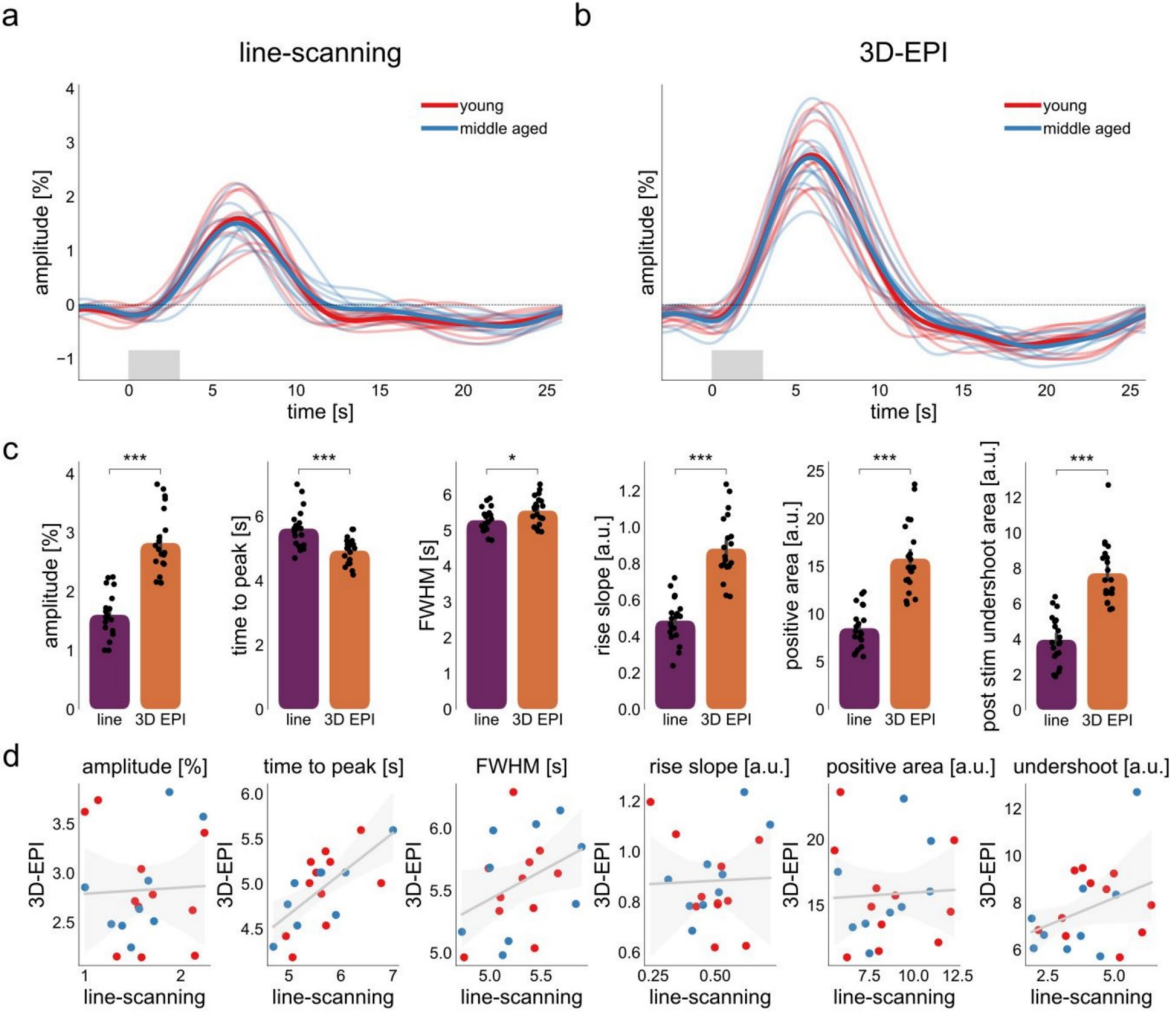
**a** Extracted HRF from line-scanning data averaged across cortical depth. **b** Extracted HRF from pV1. In both **a** and **b**, single subject HRF profiles are depicted with thin lines, and group averages are shown with thick lines for both groups. The light gray box indicates the period when the task was shown. **c** Comparison between parameter estimates extracted from line-scanning (purple) and 3D-EPI (orange). **d** Correlation between parameters extracted from line-scanning and 3D-EPI

To estimate the translatability of line-scanning, we compared the HRF profiles and parameters extracted from line-scanning and the 3D-EPI sequence (Fig. 7). It can be immediately appreciated that the magnitude of the HRF obtained with line-scanning (M = 1.61, SD = 0.37) is much lower compared to those obtained with 3D-EPI (M = 2.79, SD = 0.57). A paired-samples t-test confirmed that these differences were significant (t_19_ = − 8.45, p < 0.001, CI95% = [0.92,1.52], Cohen’s D = 2.62) (Fig. 7a). The same effect was found for positive area (t_19_ = − 7.84, p < 0.001, CI95% = [5.35,9.24], Cohen’s D = 2.42), post-stimulus undershoot (t_19_ = − 9.60, p < 0.001, CI95% = [2.93,4.57], Cohen’s D = 2.43), rise slope (t_19_ = − 8.51, p < 0.001, CI95% = [0.3,0.49], Cohen’s D = 2.65), and FWHM (t_19_ = − 2.94, p = 0.008, CI95% = [0.08,0.46], Cohen’s D = 0.74); parameters that are highly correlated with amplitude (Fig. S5). For timing parameters that depend less on magnitude, we found significantly slower time to peaks with line-scanning (M = 5.3, SD = 0.60) compared to 3D-EPI (M = 4.94, SD = 0.40), t_19_ = 6.94, p < 0.001, CI95% = [− 0.89, − 0.48], Cohen’s D = 1.35. When directly correlating parameters derived from line-scanning and 3D-EPI sequences, we found a strong correlation for the time to peak (r = 0.67, p = 0.001, CI95% = [0.33,0.86]). Other parameters that depend on magnitude, did not translate between sequences.

### Similar Cortical Depth Dependent HRFs in pV1

**Fig. 8:**
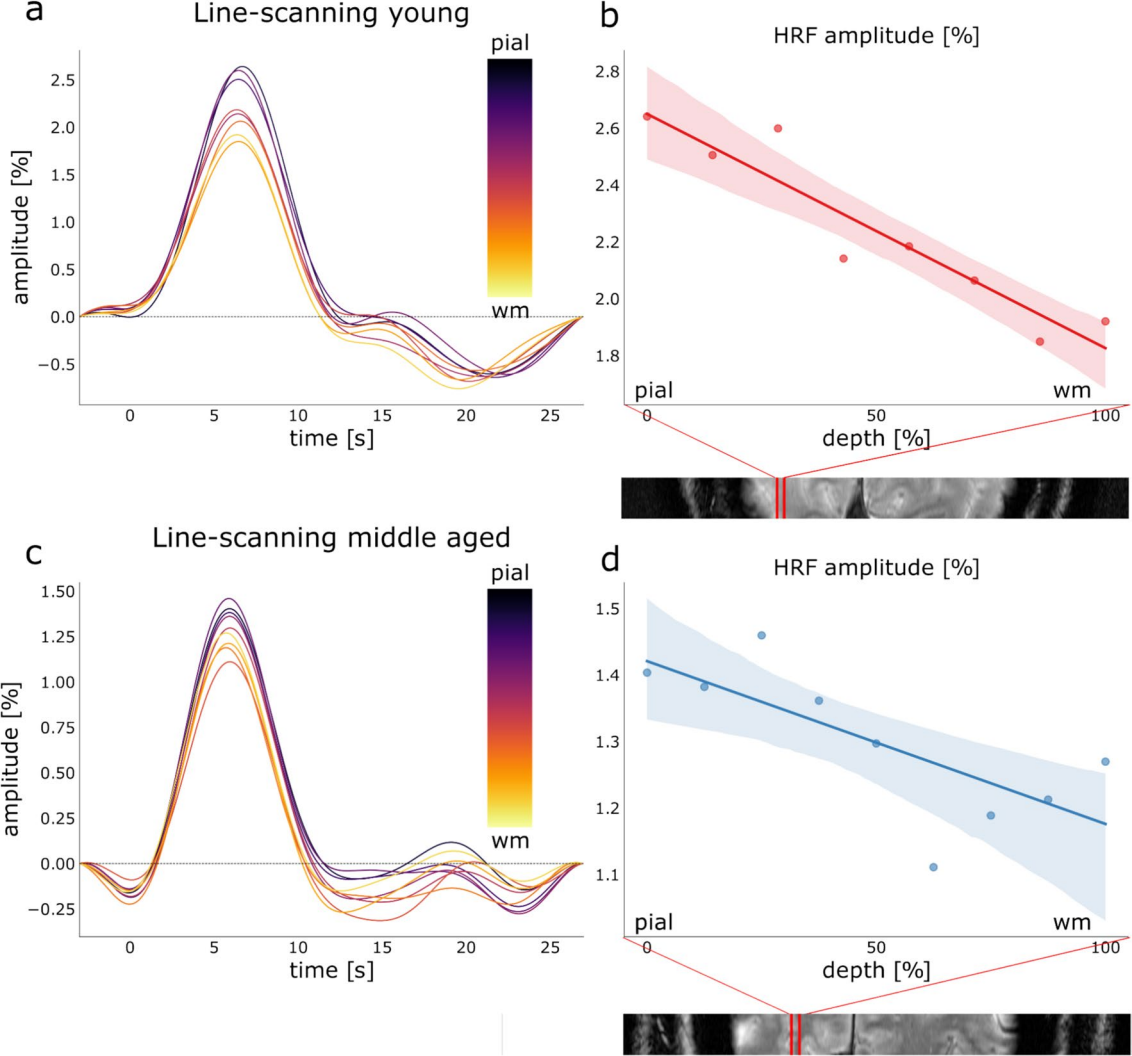
**a** Extracted HRF from line-scanning data across cortical depths for a representative young participant, and **b** corresponding amplitude across cortical depth with line ROI location reported on top of the gradient-echo slice. **c** Extracted HRF from line-scanning data across cortical depths for a representative middle aged participant and **d** corresponding amplitude across cortical depth with line ROI location reported on top of the gradient-echo slice. The fit in panels **b** and **d** is a regression fit with 95% of confidence intervals

Figure 8 shows the deconvolved HRF extracted for each cortical depth within the line ROI for a young representative participant (Fig. 8a) and a middle aged representative participant (Fig. 8c). The corresponding amplitude across cortical depth is reported in Fig. 8b, c, together with the line ROI from the gradient-echo slice acquisition.

**Fig. 9:**
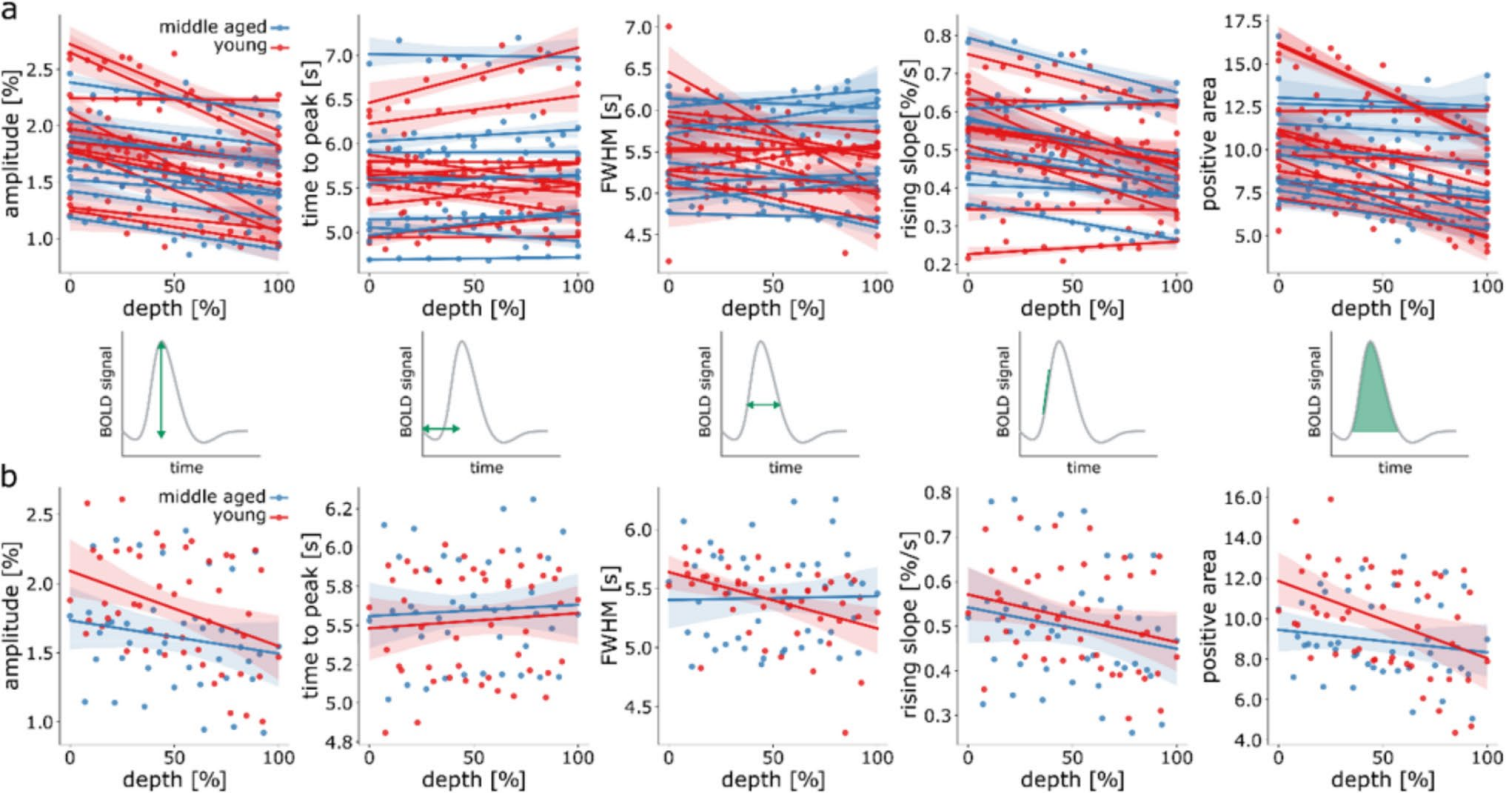
**a** HRF parameters from line-scanning data across cortical depth for all the individual participants (apart from the 2 excluded ones) and **b** for the averaged parameters across young and middle aged groups. Red indicates the young participants and blue the middle aged ones. For these plots, we calculated a linear regression between the parameter of interest and depth with a 95% confidence interval

The HRF shape across cortical depths showed similar behavior in the two age groups, with a consistent decrease in amplitude from the pial surface to the WM boundary. Note that the post-stimulus undershoot looks relatively volatile, with multiple zero-crossings between 10 and 25 s post-stimulus onset in Fig. 8. Such behavior might be expected from the deconvolution step in the line-scanning data, which are generally noisier than medium-resolution imaging data as acquired for the 3D-EPI runs. For this reason, we did not compute the post stimulus undershoot area for the HRFs across cortical depths. All the other HRF parameters were estimated across cortical depths and reported in Fig. 9a for all the individual participants and averaged across groups (Fig. 9b).

For both young and middle aged groups, the BOLD signal showed the canonical pattern of signal evolution; BOLD signals increased towards the pial surface. To test differences across depth formally, we assessed the different HRF parameters as the intercept at 50% of cortical depth and slope of the linear regression fit averaged across the 2 age groups (Fig. 9b).

For the slopes of amplitude, time-to-peak, and positive area, the assumption of normality using Levene’s test was violated (*p* < 0.001, *p* = 0.021, and *p* = 0.002, respectively). A Mann–Whitney U-test for independent samples was used to assess differences in these parameters across age groups (two-tailed, alpha = 0.025.) No corrections for multiple comparisons were made because interdependencies of parameters render the number of independent comparisons indeterminate. We found a trend for the slope of positive area (W = 79.00, *p* = 0.025), where young participants (*M* = − 0.030, *SD* = 0.020) had a steeper slope compared to middle aged participants (*M* = − 0.012, *SD* = 0.006, effect size = 0.596). No significant effects were found for the slope of the amplitude and time-to-peak (W = 75.00, *p* = 0.046 and W = 52.00, *p* = 0.882, respectively). The parameters that did not break the assumption of normality were assessed using a Student’s *t*-test (FWHM and rising slope). No significant effects were found for these parameters (t_18_ = 1.877, *p* = 0.770 and t_18_ = 0.634, *p* = 0.534, respectively). Using Bayesian independent samples t-test, we found weak evidence that the slopes of positive area, amplitude and FWHM are different across age groups (BF_10, positive area_ = 3.34, BF_10, amplitude_ = 1.604, BF_10, FWHM_ = 1.291 respectively), while for time-to-peak and rising slope showed there was weak evidence for the parameters to be identical across the two age groups (BF_10, time-to-peak_ = 0.399, BF_10, rising-slope_ = 0.459 respectively).

The difference in values at 50% of the cortical depth for all linear regressions to the shape parameters were assessed with a Student’s *t*-test. None of the intercepts at 50% depth of the parameters was significantly different in the young and middle-aged group (t_18_ = − 0.579, *p* = 0.569 for amplitude, t_18_ = − 0.339, *p* = 0.739 for time to peak, t_18_ = − 0.030, *p* = 0.977 for FWHM, t_18_ = 0.068, *p* = 0.947 for rising slope and t_18_ = − 0.278, *p* = 0.784 for the positive area). From the Bayesian independent samples *t*-test, we found weak-moderate evidence that the intercept at 50% of cortical depth for all the parameters was identical across age groups (BF_10, amplitude_ = 0.449, BF_10, time-to-peak_ = 0.415, BF_10, FWHM_ = 0.399, BF_10, rising slope_ = 0.399, BF_10, positive area_ = 0.41).

## Discussion

We did not find a significant difference in the HRF shape in the visual cortex between the young and middle aged participants. HRF changes may reflect changes in vasculature. Laminar differences in the HRF relate to the properties of the vasculature. For this reason, we also introduced a novel acquisition, i.e., line-scanning, to investigate the HRF response across cortical depth (0.25 mm spatial resolution) at an extremely high temporal resolution (0.105 s). This high sampling rate allows more detailed estimation of the HRF shape and variations of the HRF across cortical depth. Line-scanning comes at the cost of several compromises including: high spatial specificity solely in a restricted region, high noise levels and difficulties in estimating head motion. We nevertheless decided to use this technique to push the layer fMRI acquisition to its extreme, to reach the highest sampling rate and spatial resolution, which would have not been possible with a full slice acquisition and to explore future use of line-scanning acquisitions for more clinically-relevant research questions on cerebral perfusion (Van Den Brink 2024). The challenges of line-scanning were not relevant for the whole brain data acquisition (head motion estimation, isotropic resolution and SNR were not limiting). Moreover, we used our previous experiences with line-scanning data acquisition and analysis, to tackle the high noise levels (for example by using multi-echo acquisition to boost the SNR and NORDIC denoising to filter line-scanning data).

Overall, we found that there were no differences either for the HRF computed across pV1 or across cortical depth. We did find a correlation of HRF parameters for the two different sequences employed to measure the HRF across pV1 and cortical depth, in particular we found a correlation in time-to-peak of the HRF response. This suggests that the variability between participants is larger than the variability between age groups.

The HRF amplitudes derived from line-scanning data are consistent with existing literature, showing an increase in the HRF amplitude from the WM boundary to the pial surface (Siero et al. 2011). This increase is caused by the draining and pooling of blood by the upper layers and pial veins, which cause an increase of the BOLD response to the cortical surface (Markuerkiaga et al. 2016). The increases in area under the peak and steepness of the rising slope most likely also reflect this effect. Two potential confounds would be differences between the groups in the angle of the line to the cortex and differences in cortical thickness. As outlined above, the angle with the cortex was not significantly different between age groups. To assess cortical thickness (Fig. S6), we projected the image representing the nominal line to the surface. Cortical thickness values were extracted for each included intersection through the gray matter. This procedure showed that within the limited region of the line-ROI, we found no sign of cortical thinning with age (Salat et al. 2004). Thus, HRF shapes across cortical depth are consistent with previous studies, even though we did not find HRF differences with aging.

Similar HRFs as a function of aging is in accordance with previous findings that include larger sample sizes (Mayhew et al. 2022). However, finding similar HRFs in different age groups does conflict with reports that show a decrease in HRF amplitude in older age groups (Buckner et al. 2000; Ross et al. 1997; West et al. 2019). We speculate that the differences with some of the previous studies could be due to different field strengths, voxel sizes or ROI definition. First, there may be a field strength difference, as this is the first study at 7 T comparing HRF shapes in V1 in different age groups. It has been suggested that 7 T fMRI should be less selective to larger vessels (Yacoub et al. 2001). This potentially suggests there is an HRF difference with aging, but it could be driven by vascularization changes in larger vessels. Nevertheless, BOLD-weighted 7 T MRI is also sensitive to responses in larger vessels (Huber et al. 2017; Ivanov et al. 2017; Priovoulos et al. 2023). Second, voxel sizes used here were smaller than in previous studies. Although the line-scanning data benefits from high spatial resolution and reduced partial volume effects, the 3D-EPI derived ROI is of comparable size to previous work, suggesting partial volume effects to be similar.

Finally, the used ROI selection procedure may be a dominant source of variability as in most cases the ROI was based on anatomy (from standard templates) and/or function (only voxels which showed activation higher than a certain threshold). An anatomical ROI has the disadvantage that voxels are included which do not show a task response, or, also highly prevalent in the visual cortex, a negative response beyond the stimulus edge. Both will decrease the HRF amplitude and possibly distort the HRF shape (see also (Aizenstein et al. 2004) for a discussion on this regard). A functional ROI made from active voxels, i.e. based on GLM analyses, means that the voxels which best follow the GML HRF model are the same ones used to estimate the HRF shape. As in any standard GLM approach, certain assumptions on the HRF shape are made; this makes the HRF estimate based on functional ROI potentially a circular problem (Kriegeskorte et al. 2009). In our study, we combined functional anatomy and an independent functional localizer to select the voxels on which the HRF estimate was performed. Specifically, we introduced a functional localizer using a block design, which is insensitive to HRF shape and can safely be analyzed using a canonical HRF. The combination localizer scan with the probabilistic V1 based on the MNI template avoids the circularity problem on the HRF estimation and minimizes assumptions on the HRF shape.

Here, we used a task including short ISIs. Using very short ISIs can lead to an underestimation of the HRF shape if linearity is assumed. For example, Liu et al. (Liu et al. 2010), demonstrates that for ISIs shorter than 3 s, there is a notable mismatch between estimated and predicted HRFs when assuming linearity. However, with ISIs longer than 3 s, as used here, this mismatch becomes minimal. Therefore, our results are not likely due to an underestimation of the HRF shape due to very short ISIs.

Line-ROI targeting is crucial in the line-scanning experiment. Before analyzing the line-scanning results we checked the selection of a line-ROI from which to extract the HRF for the line-scanning dataset. For the line-ROI, we used multiple criteria for the voxel selection. First, we required a line perpendicular to the cortex to avoid partial volume effects across depths. We achieved an angle between the line ROI and the cortical sheet of (18 ± 12)° for the young group and (22 ± 6)° for the middle aged group. The second criterion was the proximity to pV1: we confirmed that the line-ROI was falling in the pV1 ROI or nearby for all included subjects (see Fig. S2). Finally, we avoided thin cortical patches and big veins, with the aim to exploit the high resolution of the line-scanning fMRI to detect BOLD responses from microvessels. The planning procedure could have been improved with the introduction of a separate scan session and line planning a priori to minimize partial volume effects, as suggested by Heij et al. (Heij et al. 2023). However, logistical constraints on the participant group meant that a single MRI session was preferable. Only one subject was excluded due to wrong planning, which was felt to be acceptable in this cohort (n = 22), hence we do not believe that the line-ROI targeting procedure is problematic.

Differences in participant movement may affect the results. The motion observed during the 3D-EPI acquisitions was well below the voxel size suggestion that participant movement may not affect the results. However, on average, the middle aged group moved more than the young group (Fig. S7a), which could contribute to partial volume effects in the line-scanning acquisitions. Nevertheless, these differences did not affect the cross-validated variance explained (cvR^2^) (Fig. S7b/c). We could not correct or estimate participants’ motion with the current implementation of line-scanning, but we believe that the amount of motion observed in the whole brain acquisition should be similar to the one in the line-scanning acquisition, so not leading to different results. A way to reduce motion artifacts is to introduce prospective motion correction (Andersen et al. 2019; Raimondo et al. 2023). We decided not to include motion correction in this study because the interruptions in the time series necessary to acquire motion navigators would have complicated our deconvolution approach and the T_1_ decays after every navigator acquisition would have introduced undesired signal fluctuations in the time courses, further complicating the deconvolution. Since only one subject was excluded from the line-scanning dataset due to excessive motion, we confirmed that the addition of prospective motion correction was not necessary for this study. Therefore, we do not believe that participant movement affected our results.

The performance of the perceptual tasks were similar. We used a detection task on the stimulus in order to maintain vigilance and ensure that participants’ attention was focused on the visual stimuli, all while keeping the changes to the visual stimulus minimal. From the analysis of sensitivity (d-prime) and reaction times, we concluded that there was no significant difference in the task performance between the two groups. Thus, differences in task or attention does not confound the fMRI signals.

## Conclusion

This work studied the hemodynamic response function in the visual cortex in two age groups (young and middle aged). We exploited the potential of ultra-high field MRI to increase the spatial and temporal resolutions, specifically using the line-scanning method, which allowed estimation of HRF properties also across cortical depth, in individual participants. We showed that there are no significant differences in any parameter of the HRF in the two age groups in visual cortex. This means that when non behaviorally challenging or cognitively demanding memory tasks are employed, there are no expected differences in the average HRF of cohorts including young or middle aged individuals.

## Supplementary Information

Below is the link to the electronic supplementary material.

Supplementary file1 (DOCX 1864 kb)

## Data Availability

The data and code that support the findings of this study are available from the corresponding author, upon reasonable request.

## Abbreviations

CBF: Cerebral blood flow
CBR: Cerebral metabolic rate
CMRO_2_: Cerebral metabolic rate of oxygen
MRI: Magnetic resonance imaging
fMRI: Functional MRI
BOLD: Blood oxygenation level-dependent
HRF: Hemodynamic response function
TR: Repetition time
ISI: Inter-stimulus interval
TE: Echo time
TI: Inversion time
MP2RAGE: Magnetization-prepared 2 rapid acquisition gradient echo
FLAIR: Fluid-attenuated inversion recovery
OVS: Outer volume suppression
T_1w_: T_1_-weighted
SANLM: Spatial-adaptive non-local means
CSF: Cerebrospinal fluid
WM: White matter
GM: Gray matter
GLM: General linear model
t-stats: T statistical values
FWHM: Full width at half maximum
FIR: Finite impulse response
tSNR: Temporal signal-to-noise ratio
SoS: Sum of squares
NORDIC: NOise reduction with DIstribution corrected PCA
SNR: Signal-to-noise ratio
UHF: Ultrahigh field
