# Supplementary material for: Does the Cortical-Depth Dependence of the Hemodynamic Response Function Differ Between Age Groups?
Source: Brain Topogr. 2025 Feb 28;38(3):34. doi: 10.1007/s10548-025-01107-0 (PMC11870980; doi:10.1007/s10548-025-01107-0)
Supplement: Supplementary file 1 — Supplementary file1 (DOCX 1864 kb) [file 10548_2025_1107_MOESM1_ESM.docx]

**Supplementary material**

# **
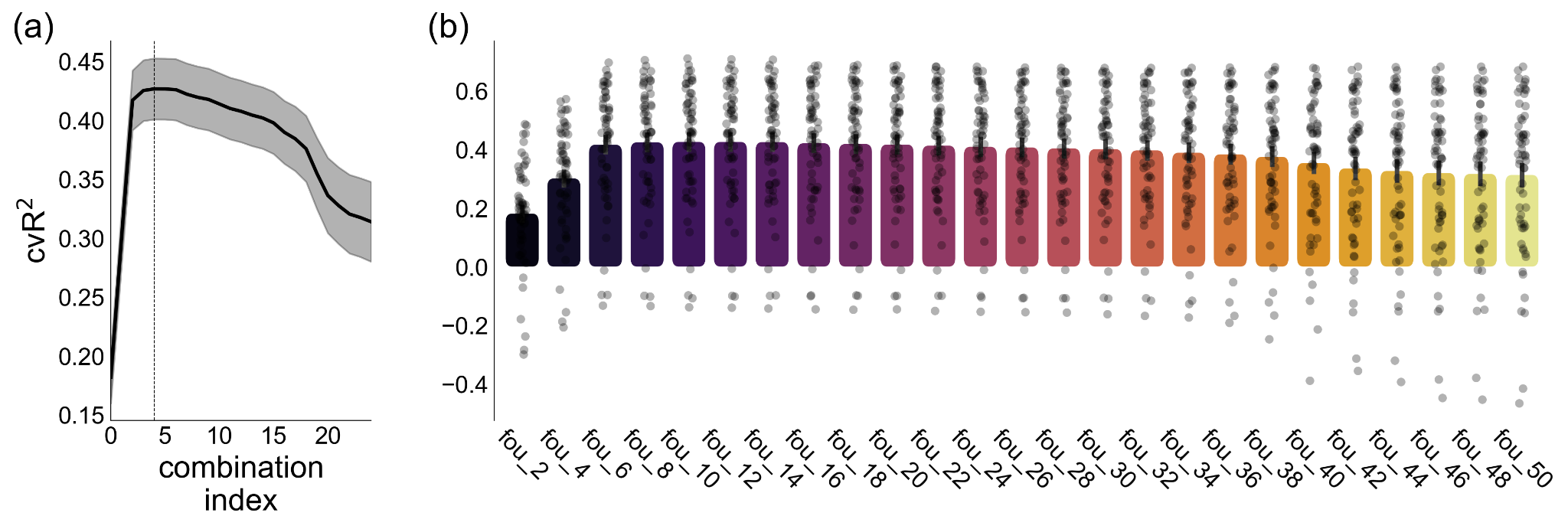
**

***Figure S1***. (a) We performed an iterative leave-one-out cross-validation for the 3D-EPI acquisition (as the events were randomized in these runs, in contrast to the fixed interstimulus intervals during line-scanning runs to boost SNR). For all possible combinations among the three runs, we deconvolved two runs and used the averaged HRF parameters to predict the non-fitted run. From this comparison, we extracted the out-of-set explained variance (cvR^2^). By looping over the 3 runs, we obtained 3 variance explained values for each subject. We repeated this approach while changing the number of regressors in the Fourier deconvolution to verify when overfitting would occur. In this dataset, the chosen deconvolution parameters (vertical line; Fourier with 10 regressors) showed the highest cross-validated variance explained. The x-axis represents different combinations, not the number of regressors. (b) Representation of (a) as bars to highlight the variation in cvR^2^ across different numbers of regressors.

# **
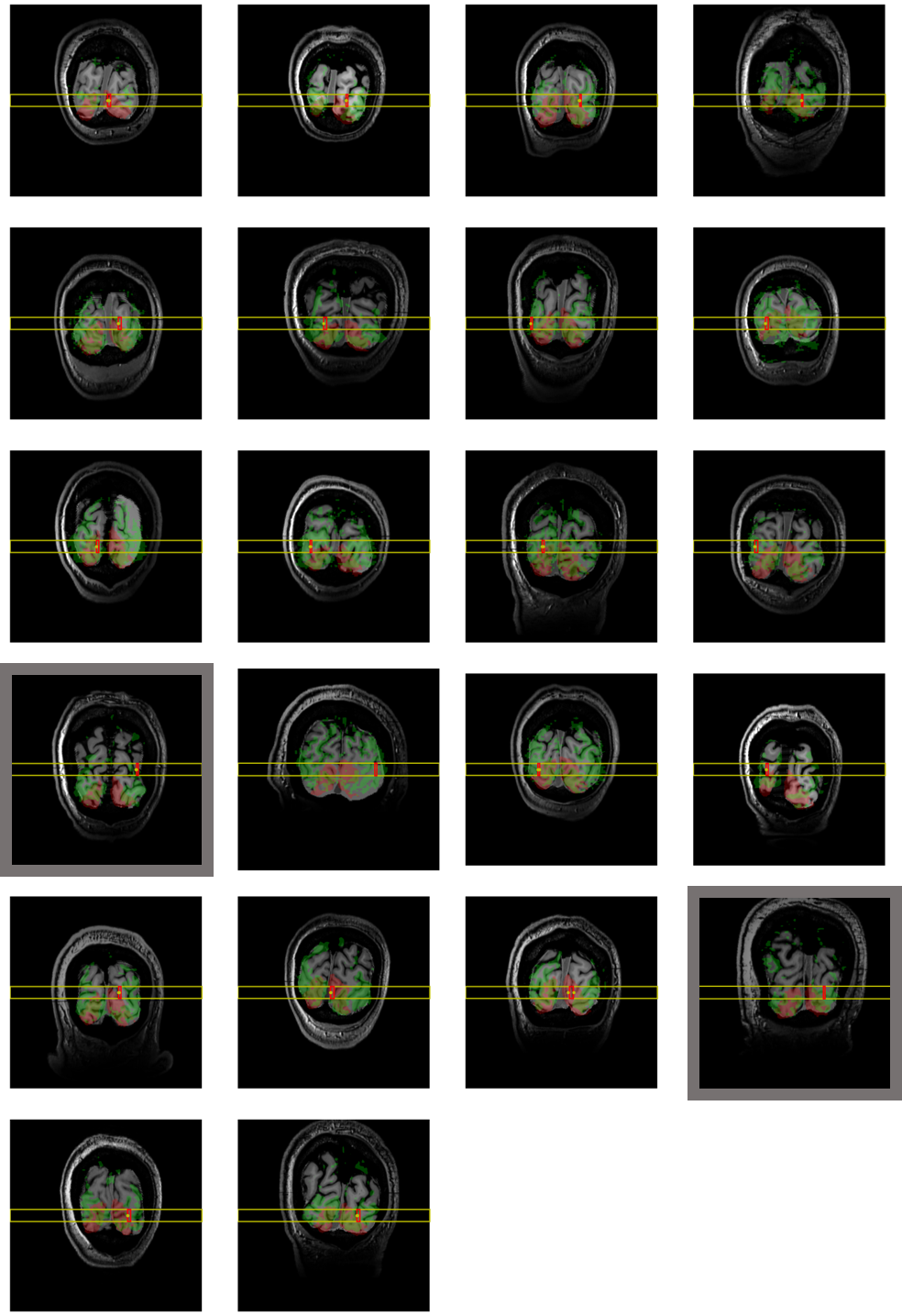
**

***Figure S2***. (a) Line-scanning planning: anatomical slice and line positioning (yellow box). Yellow dots indicate voxels perpendicular to the surface. The red box is the selected line ROI, where we estimated the HRFs across cortical depth. The Red mask is the pV1 ROI and the green mask is the localizer ROI projected on the anatomical slice. The gray boxes indicate the 2 excluded participants.


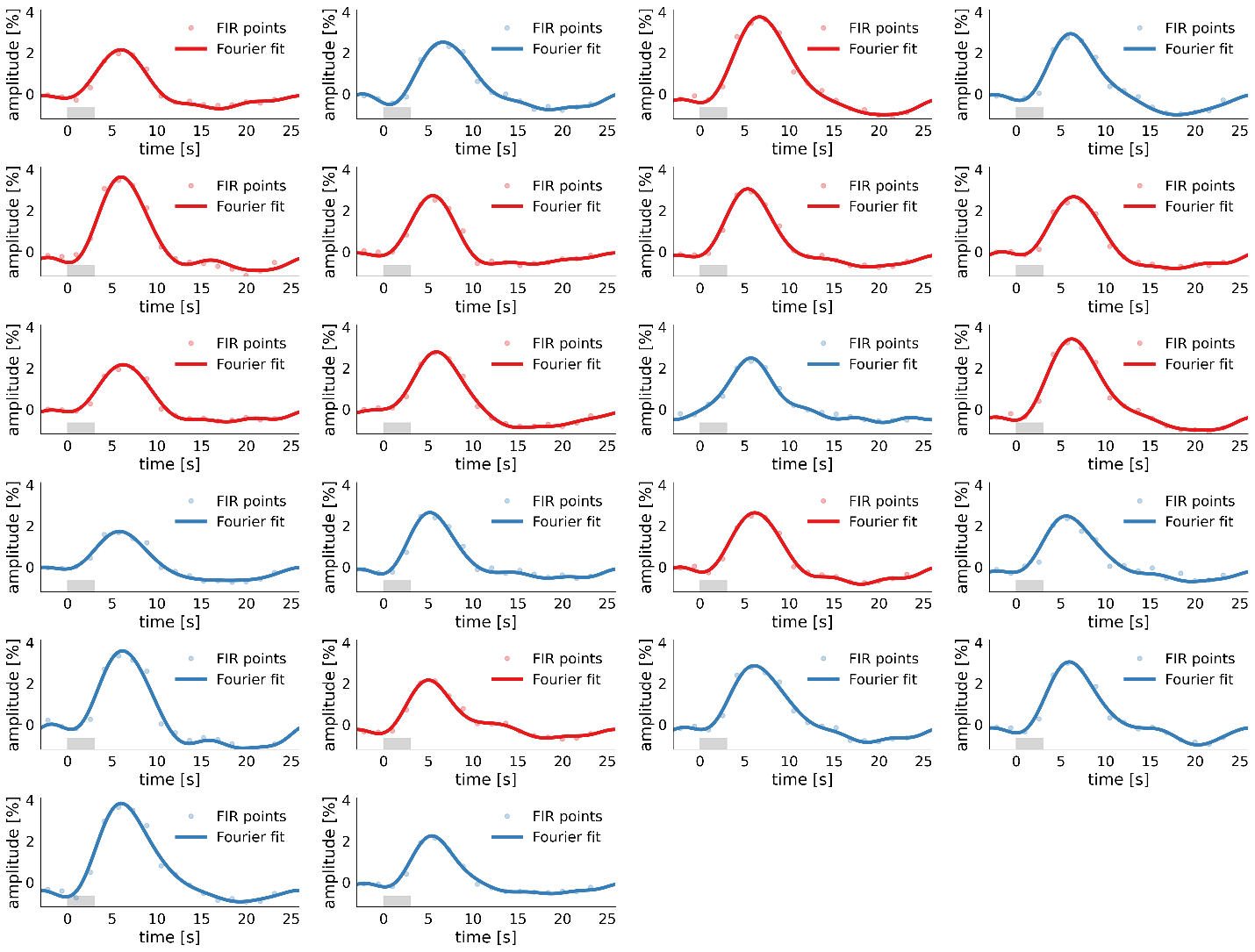


***Figure S3***. (a) Extracted HRF from whole brain data (with FIR points and Fourier basis set), for all the participants (red is used for young participants and blue for middle age participants).


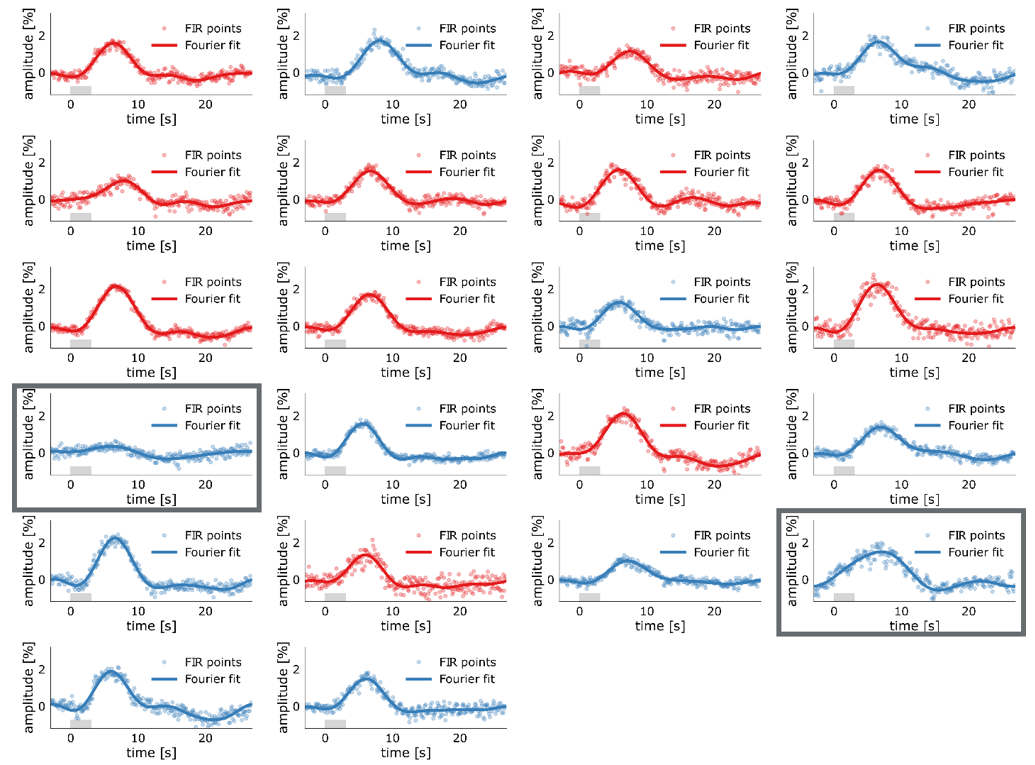


***Figure S4***. (a) Extracted HRF from line-scanning data, averaged across cortical depth (with FIR points and Fourier basis set), for all the participants (red is used for young participants, blue for middle age participants). The gray boxes indicate the 2 excluded subjects).


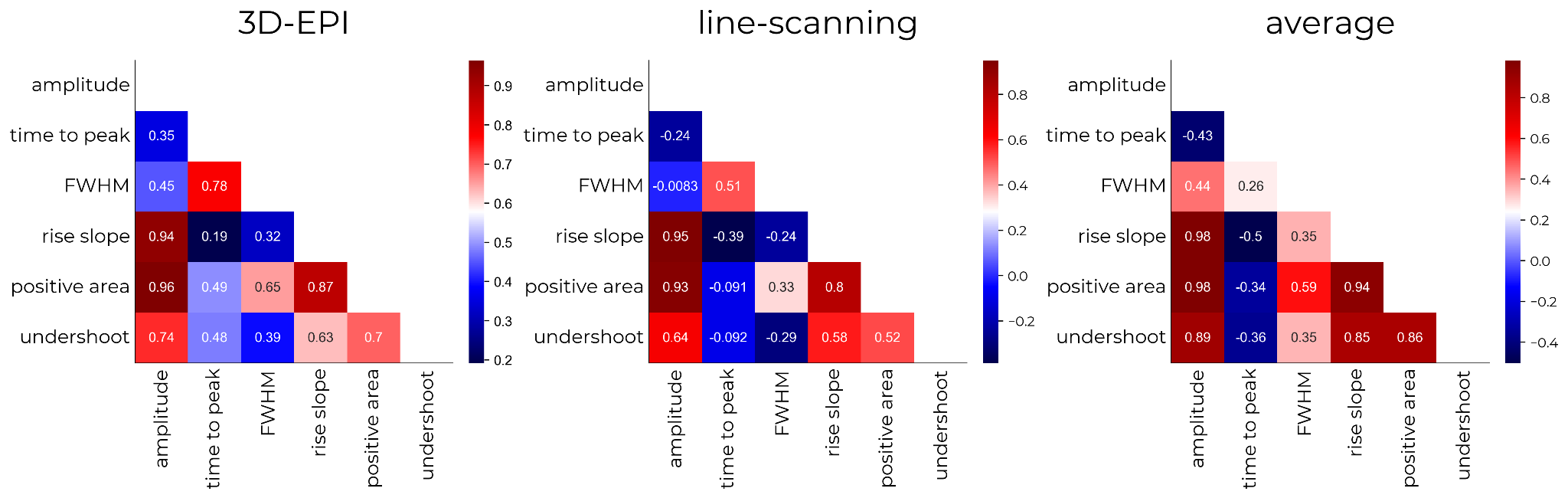


***Figure S5***. Interdependence of HRF parameters for 3D-EPI, line-scanning, and the average of both. We observe strong correlations for rise slope, positive area, and undershoot with magnitude. Timing parameters, on the other hand, do not correlate well.


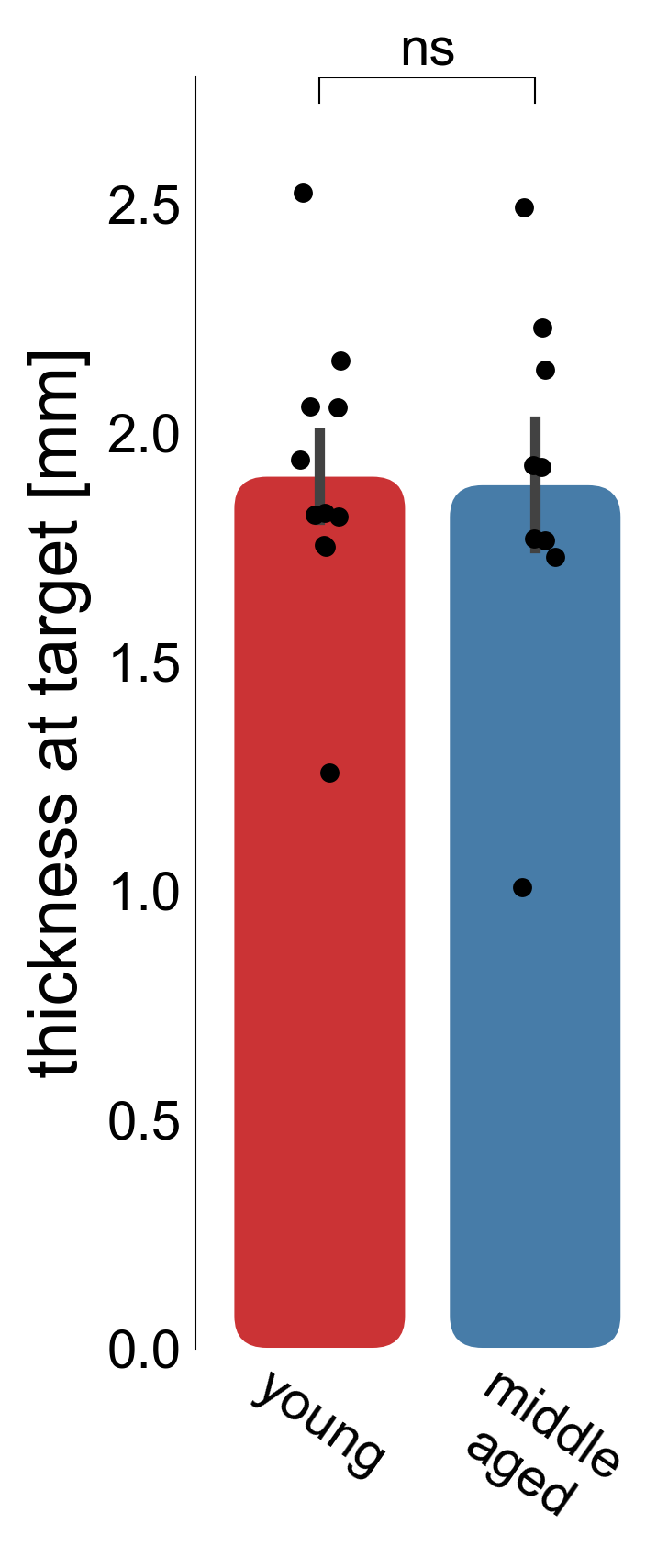


***Figure S6***. Cortical thickness at the intersection of the line with the gray matter patch did not differ between groups


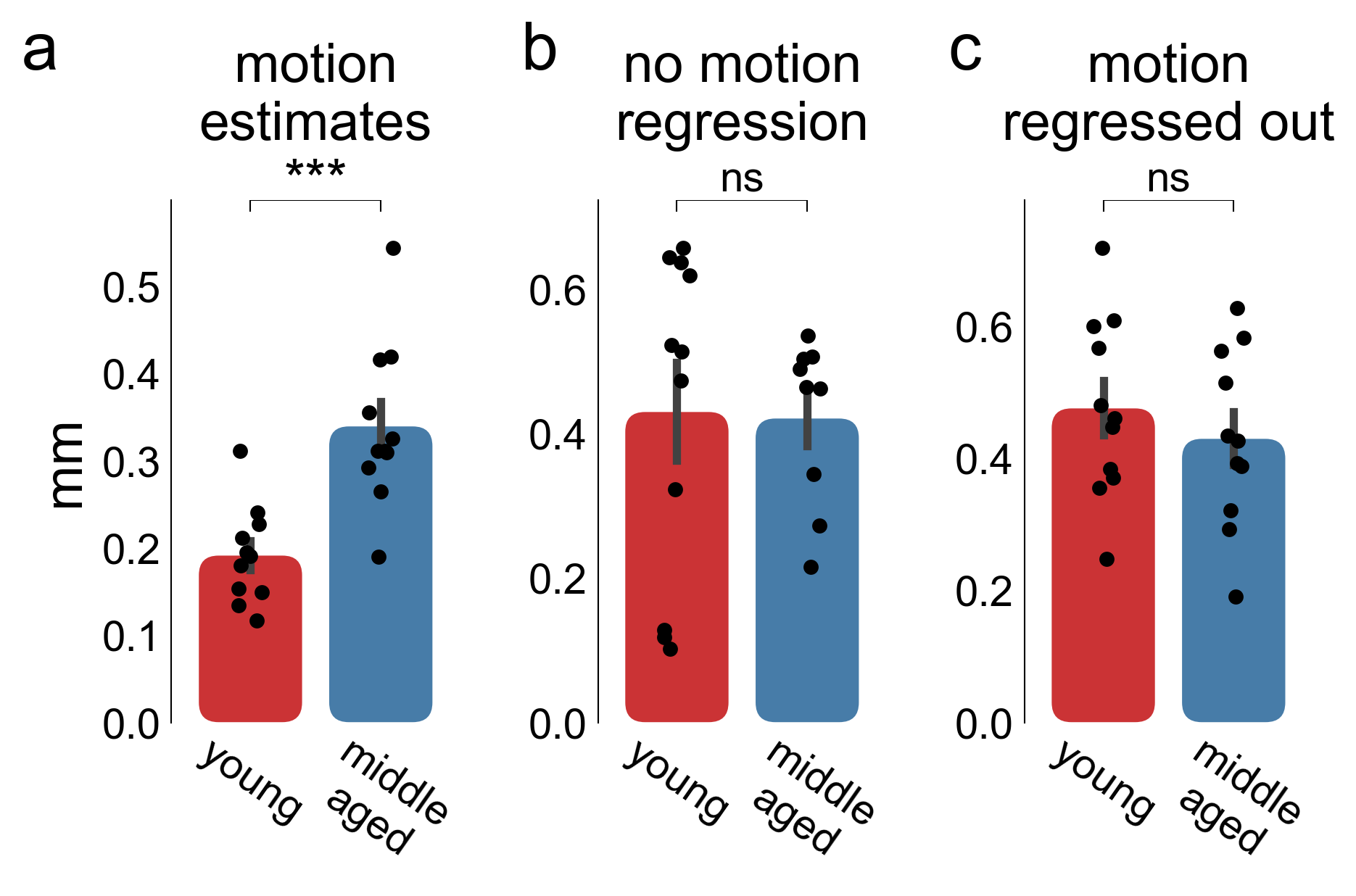


***Figure S7***. (a) Middle-aged participants moved, on average, more than younger subjects. (b) This did not affect the cross-validated variance explained (cvR2) and (c) even when accounting for motion, there is no difference in fit quality. *** = p < .001
